# Alternative diagnoses in patients referred to neuroimmunology for autoimmune encephalitis evaluation

**DOI:** 10.1007/s00415-026-13772-7

**Published:** 2026-04-01

**Authors:** Sophia F. Damman, Samhitha M. Rai, Rajeet Shrestha, Aasef G. Shaikh, Hesham Abboud

**Affiliations:** 1 Case Western Reserve University School of Medicine, Cleveland, OH USA; 2 Psychiatry Department, University Hospitals Cleveland Medical Center, Cleveland, OH USA; 3 Parkinson’s and Movement Disorder Center, University Hospitals Cleveland Medical Center, Bolwell, 5th floor, 11100 Euclid Avenue, Cleveland, OH USA; 4 Multiple Sclerosis and Neuroimmunology Program, University Hospitals Cleveland Medical Center, Cleveland, OH USA

**Keywords:** Autoimmune encephalitis, Hashimoto’s encephalopathy, PANDAS, PANS, Somatic symptom disorder

## Abstract

**Objective:**

To evaluate alternative diagnoses in patients referred to neuroimmunology for evaluation of autoimmune encephalitis (AE) and/or positive neural antibodies.

**Background:**

With increased awareness of AE, AE misdiagnosis has increased—often from improper suspicion of AE or misinterpretation of clinically irrelevant neural antibodies.

**Methods:**

We retrospectively evaluated all cases referred to our center for AE evaluation and/or a positive neural antibody. We evaluated the frequency and characteristics of patients eventually diagnosed with an alternative diagnosis.

**Results:**

A total of 119 patients were referred between 2017 and 2024. Twenty-two were referred for a positive neural antibody, and seven for possible antibody-negative AE after testing negative before referral. Eighty-one patients1 were tested by our center after inpatient admission or outpatient referral. Our center deemed antibody testing unnecessary in 9 patients. Overall, 74 patients were antibody-positive (62%). An alternative diagnosis was found in 60 patients (50.4%), including 32 with positive neural antibodies, and 28 antibody-negative patients. Of patients with alternative diagnoses, 22 had low-clinical-relevance antibodies: low-titer GAD65 (12), AchG (6), VGCC (5), and double-seronegative VGKC (4). Conversely, 10 had antibodies classically considered highly clinically relevant: high-titer GAD65 (4), GABA-BR (2), NMDAR (1), LGI-1 (1), CASPR2 (1), and GFAP (1). Of these, two had concurrent low-titer GAD65.

The most common alternative diagnoses included other immune-mediated disorders (28.3%), somatic symptom disorder (23.3%), primary psychiatric disorders (11.7%), metabolic encephalopathy/myoclonus (5%), neurodegenerative disorders (5%), and at 3.3% each, Down syndrome regression disorder, genetic disorders, neuromuscular disorders, and posterior reversible encephalopathy syndrome.

**Conclusion:**

Alternative diagnoses are common in patients referred for AE evaluation and include mostly psychiatric and other autoimmune conditions. Alternative diagnoses are not restricted to patients with low-clinical-relevance neural antibodies–they are also seen in patients with high-clinical-relevance antibodies and antibody-negative patients.

**Supplementary Information:**

The online version contains supplementary material available at 10.1007/s00415-026-13772-7.

## Introduction

Autoimmune encephalitis (AE) is a non-infectious, immune-mediated inflammation of the cortical or deep gray matter, with or without inflammation of the white matter, meninges, and spinal cord [1, 2]. While many neural autoantibodies are associated with certain subtypes of AE, some AE cases are antibody-negative, without any currently known autoantibodies detected in the serum or cerebrospinal fluid (CSF) [2]. With the increased awareness of AE, there has also been an increase in AE misdiagnosis [3]. This usually results from improper suspicion of AE or misinterpretation of clinically irrelevant neural antibodies [3, 4]. Other factors associated with misdiagnosis include incomplete antibody testing [3], misinterpretation of brain imaging [5, 6], insidious symptom onset [4], and workup lacking brain MRI and/or CSF analysis [3]. Misdiagnosis can lead to inappropriate exposure to immunotherapies with resultant patient harm and can delay time to correct diagnosis and appropriate treatment [4]. An important cornerstone in the diagnosis of AE is ruling out alternative conditions. Previous studies have identified AE mimickers in adults to include functional neurologic disorders, neurodegenerative diseases, primary psychiatric diseases, cognitive deficits from comorbidities, and atypical brain tumors [4, 6].

Only a few studies evaluated alternative diagnoses in patients suspected to have AE and either included patients evaluated prior to the publication of the 2016 AE diagnostic criteria [1, 4] or analyzed published case reports in the literature [6]. Cohorts reported were also evaluated in multiple institutions with varied levels of experience and different diagnostic approaches/thresholds to the AE diagnosis. Moreover, these studies focused mostly on the misdiagnosis of AE rather than the spectrum of patients referred for AE evaluation, which are similar but not identical concepts. In this context, we sought to evaluate the alternative diagnoses in patients referred to a specialized AE clinic in the era of increased patient and clinician awareness of AE and autoimmune neurology, and who were evaluated systematically according to modern AE diagnostic criteria and best practices [1, 2, 7]. Our goals were to determine the proportion of patients ultimately diagnosed with conditions other than AE, identify the most common alternative diagnoses in those patients, and describe their clinical characteristics.

We previously evaluated the clinical relevance of neural antibodies in patients with suspected autoimmune encephalitis and related conditions [8]. However, with emerging literature suggesting that antibody-negative AE comprises between one third to one half the cases of AE [9, 10], our AE referrals are no longer restricted to patients testing positive for neural antibodies. Many patients are now referred for evaluation of AE before being tested for neural antibodies or even after testing negative for neural antibodies prior to referral. Moreover, the increased public awareness of AE has led to a surge in the number of self-referrals. Therefore, the aim of the current study was to evaluate alternative diagnoses in all patients referred for AE evaluation regardless of their antibody status.

## Methods

Patients over age eighteen referred to the Autoimmune Encephalitis Clinic at University Hospitals Cleveland Medical Center from 2017 to 2024 for evaluation of AE and/or a positive neural antibody were included in this retrospective study of prospectively acquired data. Data was collected between 2017 and 2025. This case series was approved by the Institutional Review Board of University Hospitals Cleveland Medical Center. Patient consent was waived due to the retrospective and anonymous nature of the study. While developing the manuscript for this research, the Strengthening the Reporting of Observational Studies in Epidemiology (STROBE) reporting guidelines were adhered to.

Retrospective chart review was conducted in the electronic medical record (EMR) for all patients for medical history, clinical presentation, laboratory and imaging results, reason for referral or presentation, and final diagnosis. For the purposes of this study, previously diagnosed neoplasms included remote cancers. When continuous variables were categorized, the following boundaries were used: CSF pleocytosis was defined as white blood cells > 5, CSF protein elevation as protein > 45 mg/dL, IgG index elevation as IgG index > 0.66, C-reactive protein (CRP) elevation as CRP > 1 mg/dL, erythrocyte sedimentation rate (ESR) elevation as ESR > 20 mm/h, anti-thyroperoxidase (anti-TPO) antibody elevation as anti-TPO > 60 U/mL, and hyponatremia as sodium < 135 mmol/L. If the patient received immune-modulating therapies, type of therapy and response to therapy were recorded.

AE diagnostic categories: since some of the patients referred to our center are referred with negative neural antibody testing or without neural antibody testing at all, and since both true AE patients and patients without AE can either be negative for all antibodies, positive for clinically relevant antibodies, or positive for clinically irrelevant antibodies, we have classified the patients into the following categories:

1. True AE with positive clinically relevant antibody: patients who were designated as true AE by our team and met the Graus criteria for possible AE and/or have an Antibody Prevalence in Epilepsy and Encephalopathy (APE2) score equal to or higher than 4 + positive clinically relevant neural antibody in serum or CSF.
2. True AE with positive clinically irrelevant antibody: patients who were designated as true AE by our team and met the Graus criteria for probable or definite AE and/or met APE2 criteria for probable autoimmune encephalopathy + positive clinically irrelevant neural antibody in the serum and/or CSF.
3. True AE with negative antibody: patients who were designated as true AE by our team and met the Graus criteria for antibody-negative but probable AE or definite limbic encephalitis and/or met APE2 criteria for probable autoimmune encephalopathy + negative neural antibodies in the serum and/or CSF.
4. Not AE with positive clinically relevant antibody: patients who were designated as not AE by our team and did not meet Graus or APE2 criteria for possible AE but tested positive for antibodies considered to be clinically relevant.
5. Not AE with positive clinically irrelevant antibody: patients who were designated as not AE by our team and did not meet Graus or APE2 criteria for probable AE but tested positive for clinically irrelevant antibodies.
6. Not AE with negative or no neural antibodies: patients who were designated as not AE by our team, did not meet Graus or APE2 criteria for probable AE, and tested negative for neural antibodies or neural antibody testing was deemed unindicated by our team given very low likelihood of AE based on their low APE2 score and not meeting Graus criteria for possible AE.

Testing was required in the serum for patients to be classified as seronegative. The following neural antibodies were considered to be of low clinical relevance: AchG, VGKC without concurrent LGI-1 or CASPR2 antibody positivity (double-seronegative VGKC), striational antibodies, VGCC (unless the patient presented with ataxia), and low-titer GAD65 (GAD65 < 20 nmol/L) (Table 1). The following antibodies were considered of high clinical relevance: NMDAR, LGI-1, CASPR2, AMPAR, GABA-AR, GABA-BR, DPPX, glycine, GFAP, high-titer GAD65, Hu, Ri, Yo, CV2/CRMP5, Ma1/2, KLHL11, and amphiphysin antibodies. All neural antibodies were tested at the Mayo Clinic Neuroimmunology Laboratory. Neuronal Autoantibody Confidence Scale (NACS) Scores [2, 8] were calculated for all patients with any positive neural antibody, regardless of final diagnosis or specific antibody type. All patients deemed to have true AE met the 2016 Graus et al. AE criteria or the Antibody Prevalence in Epilepsy and Encephalopathy score for definite or probable AE. Patients with anti-TPO antibodies and clinical picture consistent with Hashimoto’s encephalopathy according to the Graus 2016 criteria and young adults with dopamine receptor antibodies and picture consistent with pediatric autoimmune neuropsychiatric syndrome (PANS) according to the 2013 PANS Consensus Conference were considered to have alternative autoimmune diagnoses for the purpose of this study [11]. Patients with alternative diagnoses were compared to those with true AE to identify early predictors of an alternative diagnosis.

**Table 1:** Examples of Patient Categorization

Patient Diagnosis	VGKC Pattern	NACS Autoantibody Component Score	Patient autoantibody test result described as:
Not Autoimmune Encephalitis	VGKC with LGI-1 and/or CASPR2 antibodies	1	Not AE with clinically relevant antibody
	Double seronegative VGKC	0	Not AE with clinically irrelevant antibody
True Autoimmune Encephalitis	VGKC with LGI-1 and/or CASPR2 antibodies	1	True AE with clinically relevant antibody
	Double seronegative VGKC	0	True AE with clinically irrelevant antibody

Patient Diagnosis	GAD Titer	NACS Autoantibody Component Score	Patient autoantibody test result described as:
Not Autoimmune Encephalitis	GAD65 ≥ 20	1	Not AE with clinically relevant antibody
	GAD65 < 20	0	Not AE with clinically irrelevant antibody
True Autoimmune Encephalitis	GAD65 ≥ 20	1	True AE with clinically relevant antibody
	GAD65 < 20	0	True AE with clinically irrelevant antibody

### Statistical analysis

After patients were organized into groups of True AE and Not AE, continuous variables were compared via t-test or, when appropriate, analysis of variance (ANOVA). For ANOVA tests with significant results, further pairwise analysis was conducted using the Tukey honest significant difference (HSD) test. Categorical variables were compared with chi square tests if 100% of calculated expected frequencies were greater than 5, otherwise Fisher’s exact test was used. For Fisher’s exact tests comparing three or more groups that showed significant differences, pairwise comparisons with Fisher’s exact test and Bonferroni correction were used for further analysis. An alpha level of 0.05 was used, except when adjusted by Bonferroni’s correction. A scatter plot was made to visually demonstrate the NACS score distribution among patients with any positive neural antibody, divided into the groups True AE with clinically irrelevant antibody, True AE with clinically relevant antibody, and Not AE with positive antibody.

## Results

### Demographics and patient presentation

**Fig. 1:**
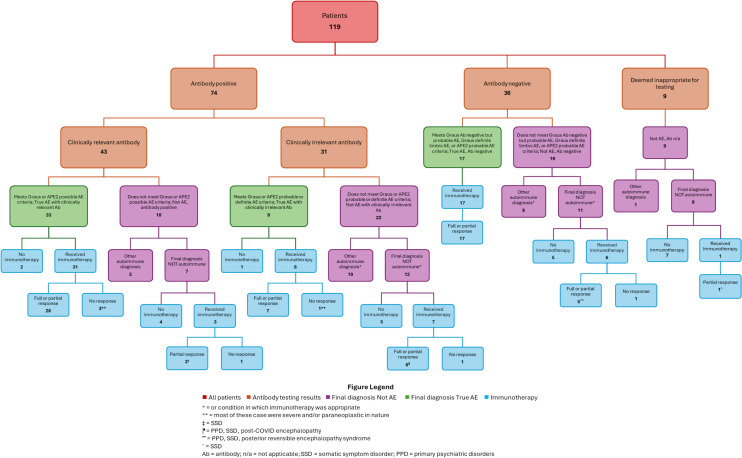
Flowchart of patients included in the current study, organized by antibody status, final diagnosis category, and treatment with or without immunotherapy. (Graphic made using Microsoft PowerPoint)

Between 2017 and 2024, 119 patients were referred for evaluation of AE or positive neural antibodies (Table 2) (Fig. 1). Nineteen (16%) were self-referred, 34 (28.6%) were physician-referred, and 66 (55.5%) were seen following hospital discharge or while inpatient. The average age was 52 (standard deviation 19.9), and 56.3% were assigned female at birth. Of all patients evaluated, 42.9% had current or prior smoking history, 29.4% had a history of autoimmune disease, and 33.6% had a neoplasm (including relevant benign tumors such as teratoma) before or during AE workup.

**Table 2:** Demographics

	Format: N (%) or Age (Standard Deviation)	All Referred Patients *N* = 119	Autoimmune Encephalitis *N* = 59	Alternative Diagnoses *N* = 60	*p* Value	Test Used
*Demographics*	*Mean age (Years)*	52 (19.9)	57 (18.3)	47 (20.3)	**0.0028**	t-test
	*Assigned female at birth*	67 (56.3)	31 (52.5)	36 (60.0)	0.4122	χ2
*Race*	*Black/African American*	24 (20.2)	15 (25.4)	9 (15.0)	0.0587	2 × 4 FET^a^
	*White*	87 (73.1)	38 (64.4)	49 (81.7)	0.0587	2 × 4 FET^a^
	*Other*	4 (3.4)	2 (3.4)	2 (3.3)	0.0587	2 × 4 FET^a^
	*Unknown*	4 (3.4)	4 (6.8)	0 (0.0)	0.0587	2 × 4 FET^a^
*Ethnicity*	*Hispanic*	4 (3.4)	4 (6.8)	0 (0.0)	0.1112	2 × 3 FET^a^
	*Unknown*	5 (4.2)	3 (5.1)	2 (3.3)	0.1112	2 × 3 FET^a^
	*Non-Hispanic*	110 (92.4)	52 (88.1)	58 (96.7)	0.1112	2 × 3 FET^a^
*Medical* *History*	*Previous or current smoking*	51 (42.9)	31 (52.5)	20 (33.3)	**0.0343**	χ2
	*Neoplasm: Previously diagnosed or discovered in workup*	40 (33.6)	27 (45.8)	13 (21.7)	**0.0054**	χ2
	*History of autoimmune disease*	35 (29.4)	14 (23.7)	21 (35.0)	0.1773	χ2
	*History of epilepsy*	12 (10.1)	3 (5.1)	9 (15.0)	0.0725	χ2
*Presentation*	*Self-referral*	19 (16.0)	3 (5.1)	16 (26.7)	**0.0013**	χ2
	*Chronic course*	47 (39.5)	14 (23.7)	33 (55.0)	**0.0005**	χ2
	*Seizures*	36 (30.3)	26 (44.1)	10 (16.7)	**0.0011**	χ2
	*Autonomic dysfunction*	31 (26.1)	13 (22.0)	18 (30.0)	0.3222	χ2
	*Movement disorder/SPS*	56 (47.1)	28 (47.5)	28 (46.7)	0.7861	χ2
*Serum* *Studies*	*Hyponatremia*	20 (16.8)	15 (25.4)	5 (8.3)	**0.0273**	χ2
	*CRP elevation*	22/61 (36.1)	14/33 (42.4)	8/28 (28.6)	0.2615	χ2
	*ESR elevation*	21/51 (38.2)	13/29 (44.8)	8/26 (30.8)	0.2840	χ2
	*Anti-TPO antibody positive*	21/41 (51.2)	6/13 (46.2)	15/28 (53.6)	0.6584	χ2
*Lumbar Puncture*	*Pleocytosis*	38/87 (43.7)	29/48 (60.4)	9/39 (23.1)	**0.0005**	χ2
	*Positive oligoclonal bands*	20/67 (29.9)	17/35 (48.6)	3/32 (9.4)	**0.0005**	χ2
	*IgG index elevation*	17/62 (27.4)	11/32 (34.4)	6/30 (20.0)	0.2048	χ2
	*CSF protein elevation*	45/82 (54.9)	31/44 (70.5)	14/38 (36.8)	**0.0023**	χ2
*Antibody Testing*	*Any positive neural antibody*	74 (62.2)	42 (71.2)	32 (53.3)	**0.0450**	χ2
	*Positive neural antibody of clinical significance*	43 (36.1)	33 (55.9)	10 (16.7)	** < 0.001**	χ2
*Imaging*	*Abnormal brain MRI*^*b*^	54/107 (50.5)	34/55 (61.8)	20/52 (38.5)	**0.0157**	χ2
	*EEG abnormalities*^*c*^	33/77 (42.9)	22/42 (52.4)	11/35 (31.4)	0.0643	χ2

χ^2^, Chi-square test; FET, Fisher Exact Test; SPS, Stiff person syndrome

Bold font, Statistically significant (*P* < 0.05)

^a^P-value determined for the entire category using the Freeman–Halton extension of the Fisher exact probability test. No additional analysis was conducted for individual groups as P-values were greater than 0.05

^b^Abnormal Brain MRI includes: hyper-intensity, contrast enhancement, DWI changes, atrophy, and edema. This does not include isolated small vessel disease or clearly incidental/unrelated findings

^c^Abnormal EEG includes: focal slowing, PLEDS, or epileptic activity

Twenty-two patients were referred for a positive neural antibody while seven were referred for possible antibody-negative AE after testing negative prior to referral. Eighty-one  patients were tested by our center (both inpatient and outpatient), with 52 patients ultimately testing positive for any neural antibody. Finally, antibody testing was deemed unnecessary by our center in nine patients referred for AE evaluation, as they presented with clinical syndromes consistent with alternate diagnoses. In total, the sample consisted of 74 (62.2%) antibody-positive patients and 45 (38%) antibody-negative patients including the nine who did not undergo testing.

An alternative diagnosis was made in 60 patients (50.4%). These patients’ average age was 47 (standard deviation 20.3), and 60.0% were assigned female at birth. Of those with an alternative diagnosis, 16 were referred for a positive neural antibody, 6 were referred with a negative antibody test prior to referral, and 29 patients were tested by our center (inpatient or outpatient). As mentioned previously, 9 patients were determined not to require testing after evaluation at the AE clinic.Twenty-three  patients with alternative diagnoses were referred by physicians, 21 were seen inpatient, and 16 were self-referred.

True AE was diagnosed in 59 (49.6%) patients. Of these, 33 had antibodies of high clinical relevance, while 26 had no antibody. Nine patients were diagnosed with true AE but had antibodies of low clinical relevance. Similar to those with alternative diagnoses, 52.5% were female; there was no sex difference between the two groups. In the True AE group, the average age was 57 (standard deviation 18.3), substantially older than those with alternative diagnoses (p = 0.003). Only 14 patients with AE presented with a chronic course (greater than 3 months), significantly less than the 33 patients with alternative diagnoses who presented with a chronic course (p < 0.001). Of these fourteen patients, 3 had no neural antibody detected on testing, while others had: Anti-Hu, double-seronegative VGKC, high-titer GAD65, high-titer GAD65 with double-seronegative VGKC, IgLON5, low-titer GAD65, NMDAR, NMDAR with low-titer GAD65, and LGI-1 (two patients). Of those with true AE, 6 were referred for a positive neural antibody, 1 was referred with a negative antibody test prior to referral, and 52 were tested by our center. With regard to referrals, of those with true AE, 11 were physician-referred and 45 were seen during or following a hospitalization. Three patients with true AE were self-referred, significantly lower than the self-referral rate for those with alternative diagnoses (p = 0.001). Those with alternative diagnoses had much lower rates of previous or current smoking than those with true AE (p = 0.034). They were also less likely to have a history of neoplasm (p = 0.005). There was no significant difference between the two groups in the frequency of pre-existing autoimmune disorders. On clinical presentation, 44% of patients with true AE had seizures, while only 16.7% of those with alternate diagnoses did (*p* = 0.001), and there was no significant difference in the history of pre-existing epilepsy between the two groups.

### Laboratory analysis and imaging

On laboratory evaluation of the CSF, patients with true AE had a higher likelihood of pleocytosis (*p* =  < 0.001), positive OCBs (*p* < 0.001), and elevated protein (*p* = 0.002). IgG index elevation was more frequently observed in patients with AE than in those with alternative diagnoses, but the difference was not significant. On analysis of serum, CRP elevation, ESR elevation, and anti-TPO antibody were observed at similar proportions among patients ultimately diagnosed with AE and those with alternative diagnoses. Conversely, patients with AE were much more likely to experience hyponatremia during their disease course (*p* = 0.027). Patients with true AE were more likely to have any positive neural antibody (71% vs 53%, *p* = 0.045) including antibodies with high clinical relevance (56% vs 17%, *p* < 0.001). Abnormal brain MRI findings were significantly more frequent in those with true AE (*p* = 0.016). While 20 patients with alternative diagnoses had abnormal MRIs, only 9 had encephalitic-like changes. Focal EEG abnormalities were observed more often in those with true AE but the difference was not statistically significant.

### Alternative diagnoses

Table 3 shows details for all patients with alternative diagnoses, including those who tested positive for any neural antibody. Overall, primary neuropsychiatric disorders were the most common alternative category, including predominantly somatic symptom disorder (23.3%) followed by schizophrenia/schizoaffective disorder (6.7%), and bipolar disorder (3.3%). The second most frequent category was other immune-mediated conditions that are related to AE, including Hashimoto’s Encephalopathy (11.7%), pediatric autoimmune neuropsychiatric disorders associated with streptococcal infections (PANDAS)/PANS (3.3%), Down syndrome regression disorder (DSRD) (3.3%), and new-onset refractory status epilepticus (NORSE) (1.7%).

**Table 3:** Case Details for Alternative Diagnoses

Category	Diagnosis	High clinical relevance antibodies	Low clinical relevance antibodies	Positive in Serum?	Positive in CSF?	Highest Titer Recorded
Neuro-psychiatric	Bipolar disorder	None	VGKC^a^	Yes	Not Done	0.08
	Bipolar disorder	None	None	NA	NA	NA
	Schizophrenia spectrum disorders	None	GAD65 Low Titer	No	Yes	0.04
	Schizophrenia spectrum disorders	None	GAD65 Low Titer	Yes	Not Done	0.17
	Schizophrenia spectrum disorders (2 patients)	None	None	NA	NA	NA
	Somatic symptom & related disorders	**GABA-B**	GAD65 Low Titer	Yes	Not Done	NA
	Somatic symptom & related disorders	**GAD65 High Titer**	None	Yes	Not Done	38
	Somatic symptom & related disorders	**GAD65 High Titer**	None	Yes	Not Done	56
	Somatic symptom & related disorders	**GAD65 High Titer**	None	Yes	Not Done	47.5
	Somatic symptom & related disorders	None	GAD65 Low Titer	Yes	Not Done	0.19
	Somatic symptom & related disorders	None	GAD65 Low Titer, VGKC^a^	Yes	Not Done	GAD65 = 0.02
	Somatic symptom & related disorders	None	VGCC, VGKC*	Yes	Not Done	VGCC = 0.04; VGKC = 0.76
	Somatic symptom & related disorders (7 Patients)	None	None	NA	NA	NA
	Unclear diagnosis, psychiatric	None	VGCC	Yes	Not Done	NA
Other Autoimmune	Amyloid beta-related angiitis	NMDAR	None	Yes	Yes	NA
	Amyloid beta-related angiitis	None	N- VGCC; P/Q-VGCC	Not Done	Yes	N-VGCC = 0.18; P/Q VGCC = 0.06
	Hashimoto's encephalopathy	None	GAD65 Low Titer, AchG	Yes	Not Done	AchG = 0.08; GAD65 = 0.11
	Hashimoto's encephalopathy	None	GAD65 Low Titer, AchG	Yes	Not Done	AchG = 0.03; GAD65 = 0.06
	Hashimoto's encephalopathy (5 Patients)	None	None	NA	NA	NA
	Neurosarcoidosis	**GABA-BR**	None	No	Yes	NA
	PANDAS/PANS	None	AchG (Transient)	Yes	Not Done	0.04
	PANDAS/PANS	None	None	NA	NA	NA
	Primary-progressive multiple sclerosis	**GAD65 High Titer**	None	Yes	Not Done	503
	Scleromyxedema	None	GAD65 Low Titer	Yes	Not Done	0.18
	Paraneoplastic transverse myelitis	None	GAD65 Low Titer	Yes	No	0.14
	Autoimmune peripheral motor neuron disease	None	None	NA	NA	NA
	Guillain–Barré syndrome	None	None	NA	NA	NA
**Genetic**	Down syndrome regression disorder	None	GAD65 Low Titer	Yes	Not Done	9
	Down syndrome regression disorder	None	None	NA	NA	NA
	Genetic ataxia	None	VGKC^a^	Yes	Not Done	0.11
	DiGeorge syndrome and Juvenile myoclonic epilepsy	None	None	NA	NA	NA
**Metabolic**	Hyperammonemia and fentanyl toxicity	**LGI-1**	None	Yes	Not Done	NA
	Metabolic encephalopathy and hospital delirium	None	AchG, VGCC	Yes	Not Done	AchG = 0.57; P/Q VGCC = 0.03
	Post-COVID-19 multifactorial encephalopathy	None	GAD65 Low Titer	Yes	No	0.22
**Neuro-degenerative**	Alzheimer Disease	**CASPR2**^b^	None	Unsure	Unsure	NA
	Idiopathic Parkinson disease	None	None	NA	NA	NA
	Posterior cortical atrophy	None	AchG	Yes	No	0.11
**Miscellaneous**	Anoxic brain Injury	None	P/Q VGCC	Yes	Not Done	0.09
	Brainstem lymphoma	None	AchG	Yes	Not Done	0.04
	Cortical myoclonus	GFAP	GAD65 Low Titer	Yes	Not Done	GFAP = 1:960; GAD65 = 0.04
	Neuromuscular disorders	None	GAD65 Low Titer	Yes	No	0.28
	Neuromuscular disorders	None	GAD65 Low Titer	Yes	Not Done	0.06
	PRES (2 Patients)	None	None	NA	NA	NA
	Post-IVIG chemical meningitis	None	None	NA	NA	NA
	Sleep deprivation	None	None	NA	NA	NA
	Idiopathic adult-onset epilepsy	None	None	NA	NA	NA
	Cryptogenic new-onset refractory status epilepticus	None	None	NA	NA	NA
	Long COVID-19	None	None	NA	NA	NA

PANDAS, Pediatric Autoimmune Neuro-psychiatric Disorders Associated with Streptococcal Infections (with positive Cunningham panel); PANS, Pediatric acute-onset neuropsychiatric syndrome; PRES, Posterior reversible encephalopathy syndrome; IVIG, Intravenous immunoglobulin

**Bold font** = High clinical relevance antibody

^a^double-seronegative VGKC

^b^positive per patient report, further specification not in chart

Of the 60 patients with alternative diagnoses, 39 received immunosuppressive therapy. Some were clinically indicated for immune-mediated disorders or conditions in which immunotherapy was appropriate. However, and remarkably, 17 patients ultimately diagnosed with non-immune-mediated conditions received immunosuppressive or immunomodulatory therapies for presumed AE that was later determined to not be AE. The most common alternative diagnoses among patients who received immunosuppressive therapy but did not have an immune-mediated condition were, in order of decreasing proportion, somatic symptom disorder (8), other primary psychiatric disorder (4), posterior reversible encephalopathy syndrome (2), anoxic injury (1), metabolic encephalopathy (1), and Alzheimer’s disease (1).

### Neural antibodies

Of patients with alternative diagnoses and positive antibodies, 10 had antibodies typically considered of high clinical relevance, including high-titer GAD65 (4), GABA-BR (2), NMDAR (1), LGI-1 (1), CASPR2 (1), and GFAP (1) antibodies. All GAD65 high-titer antibodies were detected in serum. The titers were 47.5, 56, 38, and 503 nmol/L. GABA-BR antibody was positive in the serum in one patient, and in the CSF in one patient. NMDAR antibody was positive in the serum and CSF. LGI-1, CASPR2 and GFAP antibodies were all positive in the serum.

Conversely, 22 patients with alternative diagnoses had only antibodies of low clinical relevance, including low-titer GAD65 (12), AchG (6), double-seronegative VGKC (4), and VGCC (5) antibodies. Of all patients with alternative diagnoses, 7 tested positive for 2 or more antibodies.

In addition, 41 (34%) of all referred patients were tested for anti-TPO as a part of the workup. Of the 13 patients with true AE tested for anti-TPO, 6 had elevated levels. Of the 28 patients with alternative diagnoses that were tested for anti-TPO, 15 had elevated levels but only seven of these met criteria for HE. The remaining patients were diagnosed with somatic symptom disorder, bipolar disorder, PANDAS/PANS, and DSRD.

**Fig. 2:**
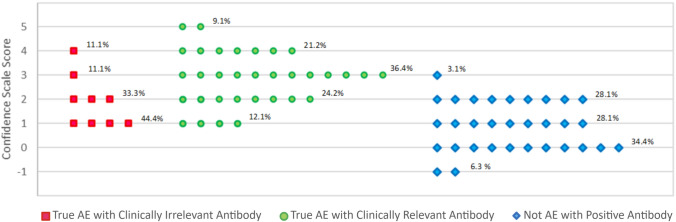
Scatter plot of NACS scores for patients with any positive neural antibody. Each point represents one patient, regardless of the number of antibodies detected on testing. (Graphic made using Microsoft Excel)

To each patient with a positive neural antibody, the NACS score was applied [2, 8] (Table 4). Patients with True AE with clinically relevant antibody totaled 33. Those with an alternative diagnosis but a positive neural antibody totaled 32. Finally, patients with true AE but with low-clinical-relevance antibodies totaled 9. The median NACS score for those with True AE with clinically relevant antibody was 3, while the median score for those with alternative diagnoses and any positive antibody was 1 (Fig. 2).

**Table 4:** Neuronal Antibody Confidence Scale Scores

	Format: N (%)	True AE—Clinically Relevant Antibodies	Alternative Diagnoses—False-Positive Antibodies	True AE—Clinically Irrelevant Antibodies	*p* value	Test Used
Number of Patients		33	32	9		
NACS Score Components	Antibody against intracellular antigen or against high clinical relevance surface antigen^a^	33 (100.0)	10 (31.3)	0 (0.0)	**0.000**	2 × 3 FT
	Movement Disorder and/or SPS	19 (57.6)	13 (40.6)	4 (44.4)	0.393	2 × 3 FT
	Neoplasm and/or Smoking	23 (69.7)	17 (53.1)	8 (88.9)	0.124	2 × 3 FT
	Inflammatory CSF^b^	18 (54.5)	5 (15.6)	5 (55.6)	**0.002**	2 × 3 FT
	Serum hyponatremia	10 (30.3)	2 (6.3)	2 (22.2)	**0.036**	2 × 3 FT
	Chronic Course (> 3 Months)	9 (27.3)	19 (59.4)	2 (22.2)	**0.018**	2 × 3 FT
Mean Total NACS Score		2.85	0.88	1.89	** <.0001**	ANOVA
Median Total NACS Score		3.00	1.00	2.00		

SPS, Stiff-person syndrome

Bold font = statistically significant (*P* < 0.05)

^a^For patients with more than one antibody identified on testing, if any of these were against an intracellular antigen or a high clinical relevance neural surface antigen, the patient received a score of 1. That is, all patients are only counted once, not separated by antibody. VGKC only considered positive if patient had concurrent evidence of CASPR2 and/or LGI-1 antibodies (i.e., double-seronegative VGKC receives 0 points)

^b^Defined as high cell count, IgG index, and/or positive oligoclonal bands; For patients without CSF testing, value assigned was 0

## Discussion

In the era of increased AE awareness among patients and clinicians, our study shows that about half of AE referrals to a specialized AE clinic have alternative diagnoses. The most common alternative diagnoses included neuropsychiatric disorders (most notably somatic symptom disorder and schizophrenia/schizoaffective disorders); and other immune-mediated disorders including AE-related conditions like Hashimoto’s encephalopathy, PANDAS/PANS, DSRD, and cryptogenic NORSE. In a modern AE tertiary clinic, referrals are not restricted to patients with positive neural antibodies as more than a third of the referrals were for antibody-negative patients. Alternative diagnoses were mainly seen in patients with neural antibodies of low clinical relevance or no neural antibodies, but an important portion had neural antibodies that are generally perceived to be of high clinical relevance including NMDAR, LGI-1, CASPR2, GABA-BR, GFAP, and high-titer GAD65. Interestingly, although positivity to NMDAR in serum alone has been linked to misdiagnosis, one of our patients with an alternative diagnosis (amyloid beta-related angiitis) was positive for NMDAR antibody in both serum and CSF, but his clinical picture was not consistent with NMDAR antibody syndrome. Clinicians should be aware of the possibility of a false positive neural antibody even in patients with antibodies of high clinical relevance but inconsistent clinical pictures. It is worth noting that none of the patients with alternative diagnoses had antibodies against intracellular antigens (the so-called classical paraneoplastic or onconeuronal antibodies), further confirming their high clinical relevance in patients evaluated for possible encephalitis [8]. Of the 33 cases of True AE with clinically relevant antibody, antibodies included NMDAR (7 patients), high-titer GAD65 (6 patients), GFAP (4 patients), Anti-Hu (3 patients), LGI-1 (3 patients), Anti-Yo (2 patients), NMDAR with GFAP (2 patients), and one patient each with high-titer GAD65, Amphiphysin, CASPR2, IgLON5, NMDAR with CV2, and anti-Yo with anti-VGCC (patient presented with ataxia) and with GFAP. Given the limited diagnostic value of neural antibodies with low clinical relevance for CNS syndromes (e.g., VGKC, AchG), it is advisable to avoid testing those antibodies in patients with suspected AE to reduce the chances of misdiagnosis.

Patients with alternative diagnoses were generally younger than AE patients and were less likely to smoke or have cancer history. Expectedly, they were less likely to present with seizures and were less likely to have abnormal CSF, hyponatremia, or encephalitic changes on MRI. Most importantly, 82.4% of self-referrals to the AE clinic ended up having alternative diagnoses. A self-referral to the AE clinic may be the most important red flag against AE.

In patients with neural antibodies, the NACS score successfully differentiated between patients with clinically relevant antibodies (True AE with clinically relevant antibody) and those with clinically irrelevant antibodies (alternative diagnoses) even when the latter group had neural antibodies classically known to be of high clinical relevance. Another important finding is that some of our true AE patients had antibodies of low clinical relevance like VGKC, low-titer GAD65, and TPO antibodies. These antibodies were likely not implicated in the pathogenesis of AE in those patients but may reflect their general propensity to autoimmunity. This group of patients had lower NACS score than patients with true AE and clinically relevant antibodies indicating that high NACS score may not only increase the confidence in the diagnosis of AE but even evaluate the relevance of the neural antibody in each individual patient. The NACS score may be a useful tool for clinicians to support clinical decision-making in patients with neural antibodies.

Our findings underscore the importance of exclusion of alternative AE diagnoses–even when other criteria have been met, and when positive neural antibodies are detected on testing. A correct diagnosis can prevent patient exposure to the harms of inappropriate immunotherapies. Moreover, reducing misdiagnosis may lower costs associated with inappropriate treatment and excessive healthcare utilization for patients and healthcare systems.

Of note, the response to immunotherapy in itself did not differentiate true AE cases from those with alternative diagnoses. This is because some true AE cases were refractory to immunotherapy, especially those that are paraneoplastic in nature or with high clinical severity. In addition, many patients with alternative diagnoses reported improvement after immunotherapy either because they had immune-mediated conditions (e.g., HE, PANDAS) or because of the general boosting effect of steroids and/or placebo effect in patients with psychiatric disorders. However, when coupled with other clinical factors suggestive of AE, the response to immunotherapy can differentiate between AE and its mimickers based on data from another study we are currently conducting (unpublished).

Compared to the multicenter study of AE misdiagnosis from 2022 by Flanagan and colleagues, our predominantly outpatient cohort with a high percentage of self-referrals had a higher rate of alternative diagnoses (50.4% vs 37%) [4]. We less frequently encountered atypical brain tumors, brain infection, or mitochondrial diseases in our clinic. We more frequently encountered other immune-mediated disorders and had similar high prevalence of somatic symptom disorder, other psychiatric disorders, and neurodegenerative disorders. The high frequency of TPO antibodies in our cohort was also seen in the multicenter study, although some of our patients did meet strict criteria for HE, which we considered an alternative diagnosis to AE. We similarly saw a high association between alternative diagnoses and low-clinical-relevance antibodies but both studies also agreed that in at least one third of antibody positive cases, patients with antibodies of high clinical relevance did not have AE including some with positive antibodies in the CSF.

Our rate of alternative diagnoses of 50% was comparable to the 46% rate of AE mimics reported by van Steenhoven et al. in their study from a national referral center in the Netherlands [5]. They also reported a high frequency of other immune-mediated disorders and psychiatric disorders comparable to our findings, but they had a much lower rate of neural antibody positivity in their mimic cohort (only 12%) including mostly low-clinical-relevance antibodies.

In a study focused on patients tested for NMDAR antibody alone, 88% had alternative diagnoses, most notably infectious, toxic, and non-inflammatory epileptic disorders, reflecting the specific clinical picture of this disorder as opposed to the general AE spectrum [12]. Expectedly, our contemporary outpatient cohort that allowed self-referrals had many more patients with psychiatric disorders compared to the meta-analysis of published case reports by Dinoto et al. [6]. The meta-analysis consisted mainly of radiologic mimickers including atypical brain tumors and genetic disorders, likely due to the publication bias of radiologically interesting cases. Our older study of clinically irrelevant neural antibodies (prior to the publication of AE diagnostic criteria and the availability of newer neural surface antibodies), which also evaluated neuromuscular patients, showed that the majority of the alternative diagnoses included other immune-mediated disorders in addition to neurodegenerative, nutritional, and psychiatric disorders [8].

One of the strengths of our study is that AE was only diagnosed when patients met probable or definite AE criteria. In addition, alternative diagnoses were carefully ruled in based on clinical and ancillary data. For example, the diagnosis of Alzheimer disease was supported clinically and with Alzheimer’s markers in the CSF.

Our study has several limitations. The final diagnosis assigned by the AE clinic was taken as the gold standard, and although all cases diagnosed as true AE met probable or definite AE diagnostic criteria, both clinical judgment and the diagnostic criteria are not immune to diagnostic inaccuracy. Some true AE and not AE patients may have been inadvertently assigned under the wrong category. In addition, most patients with alternative diagnoses did not follow at the AE clinic after the alternative diagnosis was made so we cannot confirm if new data emerged in the future that might have changed their final diagnosis. The numbers of patients with each individual neural antibody were small, so our findings represent the common alternative diagnoses of AE as a broad category but not specific to any individual antibody. Our AE clinic allows self-referrals, which might have inflated the numbers of somatic symptom disorder and other psychiatric disorders compared to clinics where self-referrals are not allowable. However, the rates of neuropsychiatric disorders in our study were not substantially different from other specialty clinic-based studies. Lastly, the NACS score lacks validation in different cohorts despite its clinical utility in our center.

## Conclusion

With the increased awareness of AE and the contemporary models of healthcare delivery that allow self-referrals, about one half of AE referrals to a specialized clinic had alternative diagnoses. Neuro-psychiatric disorders and other immune-mediated disorders of the CNS are among the most common alternative diagnoses. Self-referral was one of the strongest predictors of patients having alternative diagnoses. Those patients were also younger, were less likely to have smoking or cancer history, and were more frequently antibody-negative or had antibodies of low clinical relevance. However, about one-third of antibody-positive patients with alternative diagnoses had neural surface antibodies that are typically perceived to be of high clinical relevance. Clinicians should be critical of all neural antibodies when the clinical picture is atypical of AE regardless of the perceived clinical relevance of the antibody, its titer, or its presence in the CSF. The NACS score successfully differentiates patients with clinically relevant neural antibodies from those with clinically irrelevant antibodies and may be a useful tool to support clinical decision-making. Any new version of AE diagnostic criteria must balance the risk of over-diagnosing AE in patients with alternative diagnoses, and that of missing patients with atypical AE presentations.

## Supplementary Information

Below is the link to the electronic supplementary material.

Supplementary file1 (DOCX 18 KB)

## Data Availability

Unpublished anonymized data are available by request to any qualified investigator.
