# Supplementary material for: Alternative diagnoses in patients referred to neuroimmunology for autoimmune encephalitis evaluation
Source: J Neurol. 2026 Apr 1;273(4):239. doi: 10.1007/s00415-026-13772-7 (PMC13038682; doi:10.1007/s00415-026-13772-7)
Supplement: Supplementary file 1 — Supplementary file1 (DOCX 18 KB) [file 415_2026_13772_MOESM1_ESM.docx]

**Supplemental Materials:**

**Article title**: Alternative diagnoses in patients referred to neuroimmunology for autoimmune encephalitis evaluation

**Journal name**: Journal of Neurology (Springer)

**Author names**: Sophia F Damman, Samhitha M Rai, Rajeet Shrestha, Aasef G Shaikh, Hesham Abboud

**Affiliation and e-mail address of the corresponding author**:

Hesham Abboud, MD, PhD

Associate Professor of Neurology

Case Western Reserve University School of Medicine

Director, Multiple Sclerosis and Neuroimmunology Program

Staff Neurologist, Parkinson’s and Movement Disorders Center

Email: Hesham.abboud@uhhospitals.org

**Supplemental Table 1**: Pairwise Comparison of Mean Total NACS Score

| **Pairwise Comparison Groups** | **p Value** | **Test Used** |
| --- | --- | --- |
| True AE with clinically relevant antibody vs. Alternative diagnoses with false-positive antibodies | **p<.01** | Tukey HSD Test |
| True AE with clinically relevant antibody vs. True AE with clinically irrelevant antibody | **p<.05** | Tukey HSD Test |
| Alternative diagnoses with false-positive antibodies vs. True AE with clinically irrelevant antibody | **p<.05** | Tukey HSD Test |

Significance Thresholds: HSD[.05]=0.85; HSD[.01]=1.08

**Supplemental Table 2:** Further analysis of NACS Sub-Scores: Pairwise Analysis

| **Pairwise Comparison of Hyponatremia** | **p Value** | **Alpha level*** | **Test Used** |
| --- | --- | --- | --- |
| True AE with clinically relevant antibody vs. Alternative diagnoses with false-positive antibodies | 0.0226 | 0.0167 | 2x2 FET |
| True AE with clinically relevant antibody vs. True AE with clinically irrelevant antibody | 0.7070 | 0.0167 | 2x2 FET |
| Alternative diagnoses with false-positive antibodies vs. True AE with clinically irrelevant antibody | 0.2041 | 0.0167 | 2x2 FET |
| **Pairwise Comparison of Antibody of Significance** | **p Value** | **Alpha level*** | **Test Used** |
| True AE with clinically relevant antibody vs. Alternative diagnoses with false-positive antibodies | **< 0.001** | 0.0167 | 2x2 FET |
| True AE with clinically relevant antibody vs. True AE with clinically irrelevant antibody | 0.0830 | 0.0167 | 2x2 FET |
| Alternative diagnoses with false-positive antibodies vs. True AE with clinically irrelevant antibody | **< 0.001** | 0.0167 | 2x2 FET |
| **Pairwise Comparison of Chronic Course** | **p Value** | **Alpha level*** | **Test Used** |
| True AE with clinically relevant antibody vs. Alternative diagnoses with false-positive antibodies | **0.0125** | 0.0167 | 2x2 FET |
| True AE with clinically relevant antibody vs. True AE with clinically irrelevant antibody | 1.000 | 0.0167 | 2x2 FET |
| Alternative diagnoses with false-positive antibodies vs. True AE with clinically irrelevant antibody | 0.0670 | 0.0167 | 2x2 FET |
| **Pairwise Comparison of Inflammatory CSF** | **p Value** | **Alpha level*** | **Test Used** |
| True AE with clinically relevant antibody vs. Alternative diagnoses with false-positive antibodies | **0.0016** | 0.0167 | 2x2 FET |
| True AE with clinically relevant antibody vs. True AE with clinically irrelevant antibody | 0.0255 | 0.0167 | 2x2 FET |
| Alternative diagnoses with false-positive antibodies vs. True AE with clinically irrelevant antibody | 1.000 | 0.0167 | 2x2 FET |

*= with Bonferroni Correction
